# Pyridoxamine reduces inflammatory and microcirculatory abnormalities in metabolic dysfunction-associated steatohepatitis and modulates key factors in the hepatic AGE/ALE signaling pathway

**DOI:** 10.3389/fphys.2025.1736221

**Published:** 2026-01-15

**Authors:** Raquel Rangel Silvares, Beatriz Peres de Araujo, Evelyn Nunes Goulart Da Silva Pereira, Karine Lino Rodrigues, Juliana Magalhães Chaves Barbosa, Juliana Florencio da Silva, Vivian Vieira Dias da Silva, Marjo Van de aarenburg, Jean Scheijen, Kristiaan Wouters, Casper Schalkwijk, Anissa Daliry

**Affiliations:** 1 Laboratory of Clinical and Experimental Pathophysiology, Oswaldo Cruz Institute, Oswaldo Cruz Foundation, Rio de Janeiro, Brazil; 2 Cell Biology Laboratory, Oswaldo Cruz Institute, Oswaldo Cruz Foundation, Rio de Janeiro, Brazil; 3 Department of Internal Medicine, Maastricht University, Maastricht, Netherlands

**Keywords:** metabolic dysfunction-associated steatohepatitis, advanced glycation/lipoxidation end products, pyridoxamine, inflammatory, microcirculatory

## Abstract

Metabolic dysfunction-associated steatohepatitis (MASH) is an increasing public health concern for which new therapies are urgently needed. As growing evidence suggests that the advanced glycation/lipoxidation end products (AGE/ALE) pathway contribute to disease progression, we investigated how pyridoxamine modulates hepatic AGE/ALE-related signaling in a murine model of MASH, as well as its pharmacological impact on key features of MASH. C57BL/6 mice were fed either a standard diet (Control) or a high-fat, high-carbohydrate diet with 2% cholesterol (HFHC + CHOL2%) for 12 weeks. From weeks 6–12, subgroups of both diet groups received pyridoxamine (200 mg/kg/day), while the remaining mice received vehicles. Body and liver weights, blood glucose levels, adipose tissue distribution, liver histology, serum biochemistry, microcirculation, inflammatory cytokines, oxidative stress, and AGE/ALE signaling were assessed. The HFHC + CHOL2% group showed marked steatosis, inflammation, and impaired hepatic microcirculation. Pyridoxamine treatment attenuated metabolic and hepatic changes, reducing weight gain, hyperglycemia, fat accumulation, steatosis, collagen deposition, and the expression of proinflammatory cytokines associated with MASH. Pyridoxamine significantly reduced systemic levels of reactive dicarbonyls, such as glyoxal and 3-deoxyglucosone, and prevented the accumulation of fluorescent AGE/ALE and CML in both serum and liver. In addition, in the liver, pyridoxamine downregulates RAGE, CD36, and galectin-3 receptors, while upregulating detoxifying mediators, including AGE-R1 and glyoxalase-1. In this context, the metabolic and hepatoprotective effects of pyridoxamine appear to be associated with a rebalancing of key components of the AGE/ALE signalling pathway, potentially attenuating the toxic feedback loop that contributes.

## Introduction

Metabolic dysfunction-associated steatohepatitis (MASH), the progressive form of metabolic dysfunction-associated steatotic liver disease (MASLD), has emerged as a major global public health problem due to its continuous rise and significant clinical and economic burden (De et al., 2024; Chan et al., 2023). The pathophysiology of MASH is multifactorial and involves complex metabolic, inflammatory, and fibrogenic mechanisms (Li et al., 2024). Key mechanisms driving disease progression include lipotoxicity, persistent immune activation, and redox imbalance, which exacerbate cellular dysfunction and chronic liver injury (Tilg and Moschen, 2010).

The pathway of advanced glycation and lipoxidation end products (AGE/ALE) has been extensively studied and has been shown to play a role in the development of various chronic diseases, including diabetes mellitus, cardiovascular disease, chronic kidney disease, and neurodegenerative disorders (Ramasamy et al., 2011; Zhou et al., 2024; Wang et al., 2024). AGE and ALE are heterogeneous groups of molecules formed through non-enzymatic post-translational modifications of proteins, lipids, or nucleic acids by glycation and lipoxidation, respectively (Priken et al., 2022). Their interaction with specific receptors, such as the receptor for AGE (RAGE), triggers inflammation and a redox imbalance, which disrupts cellular processes. There is evidence that AGE/ALE and their receptor-mediated effects play an important role in the pathophysiology of MASLD. Experimental models and early clinical studies, including the results of our group, have demonstrated that elevated levels of AGE/ALE correlate with the severity of steatosis, inflammation and fibrosis (Priken et al., 2022; Asadipooya et al., 2019; Bijnen et al., 2019; Pereira et al., 2021a).

Pyridoxamine, a bioactive form of vitamin B6, has been shown to inhibit the formation of AGE/ALE, limit tissue damage and prevent pathological processes (Pereira et al., 2020; Wilson et al., 2019) by quenching dicarbonyls. Pyridoxamine also acts as a cofactor in over 140 enzymatic reactions involved in amino acid, glucose and lipid metabolism (Parra et al., 2018). Previous studies by our group have shown that pyridoxamine ameliorates metabolic and microcirculatory disturbances in a MASLD model by downregulating AGE levels and TNF-α expression (Pereira et al., 2020). Furthermore, we have shown that pyridoxamine supplementation in combination with lifestyle modifications may be an effective strategy to treat metabolic and hepatic vascular complications in MASLD (Pereira et al., 2021b). In support of these findings, Kobayashi et al. (2021) reported that pyridoxamine administration improved hepatic steatosis in MASLD patients, even without significant changes in body weight or body mass index (Kobayashi et al., 2021).

Although some features of pyridoxamine’s effects on the AGE signaling pathway have been reported in the literature, few studies have examined its impact on specific points within the pathway or explored multiple mechanisms, especially in MASH-associated pathogenesis. In this study, we investigated in detail how pyridoxamine modulates the AGE/ALE pathway, including analysis of ligands, intermediates, and receptors in the liver, and evaluated its pharmacological effects on key features of MASH such as inflammation, oxidative stress, and microcirculatory dysfunction. Our results provide new insights into the therapeutic potential of inhibiting the AGE/ALE signaling pathway in the progression of MASH.

## Methods

### Ethical considerations

All experiments were conducted in accordance with internationally accepted principles for the care and use of laboratory mice and approved by the Animal Welfare Committee of the Oswaldo Cruz Foundation (Licence L-012/2018 A2).

### Experimental design

A total of 40 male C57BL/6 mice (8 weeks old) were used, all of which were kept in the central animal facility of the Oswaldo Cruz Foundation. The mice were housed in standard cages with controlled temperature (22 °C ± 1 °C) and a 12-h light/dark cycle (dark phase from 18:00). Mice in the HFHC + CHOL2% group were fed a high-fat diet (60% kcal from fat +2% cholesterol; Pragsoluções, São Paulo, RJ, Brazil) for 12 weeks and were given *ad libitum* access to a high-carbohydrate drinking solution (45 g/L; 55% fructose, 45% sucrose). The control mice received an adapted purified standard diet (10% kcal from fat; Pragsoluções, São Paulo, Brazil) and water for the same duration. A detailed composition of both experimental diets, including macronutrient sources and the functional classification of carbohydrates, lipids, proteins, fiber, vitamins, and minerals, is provided in Table 1. From the sixth to the 12th week, the two diet groups (control and HFHC + CHOL2%) were randomly divided into two treatment groups. The mice assigned to the vehicle groups continued to receive their respective diets and were orally administered the water for the last 6 weeks (n = 20). The mice in the pyridoxamine groups continued to receive the same diet and were treated with pyridoxamine (200 mg/kg/day) by oral gavage for the last 6 weeks (n = 20).

**TABLE 1:** Comparative composition of control and HFHC + CHOL2% diets.

Category	Component	Control diet (10% fat)	HFHC+CHOL2% diet (60% fat)
Proteins	Casein	18.96%	25.85%
	L-cystine	0.28%	0.39%
Carbohydrates – complex	Corn starch	47.98%	0.00%
	Dextrinized starch	11.85%	16.15%
Carbohydrates – simple	Sucrose (solid diet)	6.52%	6.89%
Lipids – unsaturated	Soybean oil	2.37%	3.23%
Lipids – saturated	Lard	0%	31.66%
Dietary fiber	Microcrystalline cellulose	4.74%	6.46%
Minerals	Dibasic calcium phosphate	1.23%	1.68%
	Calcium carbonate	0.52%	0.71%
	Potassium citrate	1.56%	2.13%
	Mineral mix (PSB10026)	0.95%	1.29%
Vitamins	Vitamin mix (AIN-76A)	0.95%	1.29%
Other components	Choline bitartrate	0%	0.26%
	Cholesterol	0%	2.00%

Animals in the HFHC+CHOL2% groups additionally received a carbohydrate-enriched drinking solution (45 g/L; 55% fructose and 45% sucrose), further increasing exposure to refined sugars.

### Fasting blood glucose

At the end of the experiment, fasting blood glucose levels were measured using the Accu-Chek Performa glucometer (Roche Diagnostics, Basel, Switzerland). Mice were fasted for 6 h, a small incision of less than 1 mm was made with surgical scissors at the tip of the tail to collect a drop of blood, which was applied to a test strip inserted into the glucometer. Glucose values were expressed in mmol/L.

### *In vivo* microcirculatory parameters

At the end of the experiment, the following parameters were analysed in the liver: microvascular blood flow, leukocyte rolling and adhesion, and vitamin A-positive cells. Mice were anaesthetised by intraperitoneal injection (ip) of ketamine hydrochloride (100 mg/kg) and xylazine (10 mg/kg). The left lobe of the liver was then exteriorised for microcirculation analysis.

1. Intravital microscopy: interaction between leukocytes and endothelial cells was assessed by counting labelled leukocytes (0.3 mg/kg rhodamine 6G, intravenous (iv), rolling or adherent to the sinusoids and postsinusoidal venules. The number of HSCs was quantified by endogenous fluorescence of vitamin A using a UV filter system. The number of vitamin A-positive HSCs rolling and adherent leukocytes was counted in an area of 170 μm^2^, which included the sinusoids and post-sinusoidal venules (Olympus BX150WI).
2. Laser speckle flowmetry: Hepatic microvascular blood flow was measured using Laser Speckle Contrast Imaging (LSCI) (Pericam PSI system, Perimed, Sweden), which provides a microcirculatory perfusion index proportional to the concentration and average velocity of erythrocytes. This system was used to measure blood flow in the microvascular bed in real time. Under anaesthesia, the mice were placed under a laser light system with an image contrast wavelength of 785 nm to continuously measure tissue perfusion. The relative hepatic blood flow of the mice was expressed in arbitrary perfusion units (APUs).

### Euthanasia and blood and tissues collection

At the end of the experimental protocol and the microcirculation analyses had been performed on the anesthetized mice, a cardiac puncture was performed for blood sampling. The blood samples were centrifuged to obtain serum, which was stored at −80 °C for further analysis. The liver and adipose tissue were removed and weighed, and the liver was also stored at −80 °C for further experiments.

### White adipose tissue (WAT)

To analyse body fat mass, the subcutaneous inguinal and thoracic WAT deposits and the visceral WAT deposits (retroperitoneal, mesenteric, and epididymal) were dissected *postmortem*. Subcutaneous WAT was defined as the deposit under the skin in the groin area. Retroperitoneal fat refers to the fat in the abdominal cavity extending from the diaphragm to the pelvis, bounded posteriorly by the transverse fascia and anteriorly by the posterior parietal peritoneum. Mesenteric WAT was defined as an accumulation of fat pads adherent to the intestines. Epididymal WAT was identified as the deposit in the lower abdomen, associated with the head of the epididymis. After dissection, the WAT deposits were weighed using an analytical balance (GR-200; A&D Company Limited, Toshima-ku, Japan). The weight of the visceral WAT was calculated by adding the weights of the retroperitoneal, mesenteric and epididymal deposits.

### Biochemical markers

Serum levels of total cholesterol, HDL, LDL, triglycerides, ALT enzyme activity, and hepatic cholesterol and triglyceride levels were measured using commercial kits (Bioclin System II, Belo Horizonte, Brazil). For the determination of total cholesterol and triglycerides in the liver, 50 mg of homogenised liver tissue was extracted in 1 mL of isopropanol and then centrifuged at 2000xG for 10 min. After centrifugation, the supernatant was separated and analysed. Results were normalised to tissue weight and expressed as mg per g of liver tissue (wet weight).

### Histopathology

Samples from the left lateral lobe of the liver were fixed in Milloning formalin (pH 7.2) for at least 48 h, processed using standard histological procedures, and then embedded in paraffin for light microscopic analysis (Nikon, Model 80i and DSRi1 digital camera, Nikon Instruments, Inc., Melville, United States). The histological features of sections stained with haematoxylin and eosin (H&E) were classified into the following categories: Steatosis, inflammation and hepatocellular injury. Each sample was analysed using the stereological point-counting method for measuring liver fat content with STEPanizer software (version 1, Berna, Switzerland). Inflammation and ballooning were analysed by examining the slides under a microscope with ×20 magnification. The NAS (Non-Alcoholic Steatohepatitis Activity Score) was used to classify the stage of liver disease as follows: MASH (score ≥5), borderline (score 3-4), or non-MASH (score 0-2). This classification was based on the sum of the scores of the variables assessed, with the disease score categorised according to the parameters described in Kleiner et al. (2005). For each parameter, five histological sections from seven mice per group were analysed. Masson’s trichrome staining was used to assess fibrosis by quantification of blue stained collagen, using ImageJ software (ImageJ version 1.53e, Madison, WI, United States) for image segmentation. Five histological sections from six mice per group were analysed for each parameter.

### Immunohistochemistry

After deparaffinisation, histological sections were incubated in a citrate buffer at 60 °C and then treated with 3% H_2_O_2_. Non-specific binding was blocked with normal horse serum (2.5%). Sections were then incubated overnight in a humidified chamber at 4 °C with primary mouse monoclonal antibodies against α-SMA (1:500, sc-53015, Santa Cruz Biotechnology, Santa Cruz, United States), RAGE (1:200, sc-365154, Santa Cruz Biotechnology, Santa Cruz, United States), and CML (1:200, ab125145, Abcam, Cambridge, United Kingdom). Slides were washed in 1x PBS and treated with a biotinylated secondary antibody, followed by incubation with streptavidin-HRP at room temperature. Diaminobenzidine (DAB) was used as the chromogen. The protocol followed the manufacturer’s recommendations (PK7800 and SK-4100, Vectastain Universal Quick kit, Peroxidase, R.T.U., Vector Laboratories, Newark, United States). Slides were counterstained with Mayer’s haematoxylin for light microscopic analysis. Quantification of immunostaining was performed using the ImageJ software (ImageJ version 1.53e, Bethesda, United States).

### Enzyme-linked immunosorbent assay (ELISA)

Quantification of CML in liver was performed using an enzyme -linked immunosorbent assay (ELISA) kit for general CML (Carboxymethyl Lysine) (ELK7896 – ELK Biotechnology CO., Denver, United States) according to the manufacturer’s instructions.

### AGE levels by fluorescence analysis

The concentrations of fluorescent AGE in liver tissue and serum were determined according to the method described by Nakayama et al. (1993). In brief, the fluorescence of the samples was measured at an excitation wavelength of 360 nm and an emission wavelength of 460 nm using a SpectraMax M5 ELISA microplate reader (Molecular Devices, San Jose, United States). A native bovine serum albumin (BSA) preparation (1 mg/mL in NaOH 0.1N) was used as a reference, and its fluorescence intensity was defined as one fluorescence unit. The fluorescence values for the samples, adjusted to a protein concentration of 1 mg/mL using the BCA Protein Assay Kit (Thermo Fisher, Waltham, United States) were expressed in arbitrary units (AU) compared to the native BSA fluorescence.

### qPCR (quantitative polymerase chain reaction)

Total RNA was isolated from the middle lobe of the liver using the RNeasy Mini Kit (Qiagen, Venlo, the Netherlands). Complementary DNA (cDNA) synthesis was synthesised using the High-Capacity cDNA Reverse Transcription Kit (Applied Biosystems, Foster City, United States) with 1 μg RNA in a final volume of 20 μL. The primers used for the amplification of the target genes are listed in Table 2. The qPCR was performed with the Power SYBR Green PCR Master Mix (Applied Biosystems, Foster City, United States) according to the manufacturer’s instructions using the 7,500 Fast platform (Applied Biosystems, Foster City, United States). The relative expression of target genes was calculated using the ΔΔCt method, normalised to β-actin expression.

**TABLE 2:** Primer sequences used for quantitative real-time PCR (qRT-PCR).

Gene	Forward primer (5’–3’)	Reverse primer (5’–3’)
TNF-α	CTACCTTGTTGCCTCCTCTTT	GAGCAGAGGTTCAGTGATGTAG
IL-6	ACAACCACGGCCTTCCCTACTT	CACGATTTCCCAGAGAACATGTG
NF-κB	GAAGTGAGAGAGTGAGCGAGAGAG	CGGGTGGCGAAACCTCCTC
RAGE	CAGGGTCACAGAAACCGG	ATTCAGCTCTGCACGTTCC
CD36	CCTGGGAGTTGGCGAGAAA	CGATCACAGCCCATTCTCCT
AGE-R1	ACCTTCAAGACCGCAGATGA	CACGTTGATGTTGCCTCCAA
Galectina-3 (GAL-3)	CCCGCATGCTGATCACAATC	GGGGTTAAAGTGGAAGGCAA
Glyoxalase (GLO-1)	CCCTGCTATGAAGTTCTCGCTC	GAGCTCAAGGGTGGCTTTTCT
DIAPH-1	TGTCACCTCTGCTTTTCCTC	GAGAGTGGTTGAGACCCTTTG
β-actin	AGATTACTGCTCTGGCTCCTAGC	ACTCATCGTACTCCTGCTTGCT

**TABLE 3:** Pyridoxamine improves systemic and hepatic metabolic parameters in mice with diet-induced MASH.

Parameters	CONTROL	CONTROL+PYR	HFHC+2%CHOL	HFHC+2%CHOL+PYR
Total Cholesterol (mg/dL)	113,57 ± 5,53	117,33 ± 11,91	211,07 ± 22,44 ***	122,89 ± 8,92 $$$
LDL Cholesterol (mg/dL)	13,53 ± 0,71	10,75 ± 1,49	22,35 ± 1,58 **	11,92 ± 2,06 $$$
HDL Cholesterol (mg/dL)	71,36 ± 3,38	80,60 ± 6,90	108,05 ± 3,07 ***	101,37 ± 6,09
Serum Triglycerides (mg/dL)	40,30 ± 4,07	26,56 ± 2,83	48,82 ± 2,88	52,28 ± 6,67
ALT (U/L)	36,25 ± 3,92	27,50 ± 13,56	98,29 ± 15,27 *	47,83 ± 13,16
Hepatic Cholesterol (mg/g)	6,60 ± 1,03	2,49 ± 0,32	19,60 ± 1,38 ***	9,65 ± 1,36 $$
Hepatic Triglycerides (mg/g)	56,75 ± 11,73	25,127 ± 2,11	106,56 ± 8,02 **	64,29 ± 7,852 $$

*P < 0.05 vs. CONTROL; **P < 0.01 vs. CONTROL; **P < 0.001 vs. CONTROL; $$ P < 0.01 vs. HFHC + CHOL2%; $$$ P < 0.001 vs. HFHC + CHOL2%.

### Ultra-performance liquid chromatography tandem mass spectrophotometry (UPLC-MS/MS)

To measure the specific AGE CML, CEL and MG-H1 and the dicarbonyls MGO, GO and 3-DG, and lysine, 25 μL of serum was used, following the protocol described by Scheijen et al. (2016). In brief, after deproteinisation and hydrolysis of the samples, they were evaporated under nitrogen until dry and then dissolved in 500 μL of water. For the measurement of lysine, CML, CEL, MG-H1, MGO, GO, 3-DG derivatives and non-derivatised lysine, 10 μL of the hydrolysate was diluted in 800 μL of water and analysed by UPLC MS/MS. The concentrations of protein-bound or free CML, CEL, MG-H1, MGO, GO, and 3-DG were determined by comparison with calibration curves. All protein-bound or free AGE fractions were expressed in mmol lysine to match the protein content in each sample.

### Flow cytometry

The Cytometric Bead Array (CBA) Mouse IL-6, INF-γ, TNF-α e IL-2 Cytokine Kit (BD, Becton Dickinson, United States) was used to analyse the cytokines present in the liver. Liver tissue samples were thawed and lysed by sonication in a phosphate buffer containing a cocktail of protease inhibitors and Nonidet P40, both at a final concentration of 1%. The samples were centrifuged at 500 *g* for 5 min, and the supernatant was collected. Then, 25 µL of the samples, 25 µL of the capture bead mixture, and 25 μL of the fluorochrome phycoerythrin were incubated for 2 h at room temperature. After washing 500 µL of wash buffer, the samples were centrifuged at 200 *g* for 5 min. The supernatant was discarded, and the samples were resuspended in a 100 µL wash buffer. Data acquisition was performed using the Cytoflex flow cytometer (Beckman Coulter, United States). Total protein determination for liver was performed using the BCA Protein Assay Kit (Thermo Fisher, Waltham, United States), following the manufacturer’s instructions for normalisation.

### Oxidative stress

Oxidative stress in liver tissue was assessed by analysing lipid peroxidation, reactive oxygen species (ROS) formation and endogenous antioxidant defence mechanisms.

#### Lipid peroxidation

Oxidative stress in the liver was assessed by quantification of thiobarbituric acid reactive species (TBARS). Samples (0.5 mL) were mixed with an equivalent volume of 0.67% thiobarbituric acid and heated at 96 °C for 30 min. The absorbance of the samples was measured at 535 nm using the SpectraMax Plus Reader (Molecular Devices, San Jose, United States).

#### Reactive oxygen species (ROS)

The formation of ROS was quantified using the 2′,7′-dichlorofluorescin diacetate (DCFH-DA) assay. Liver tissue homogenate was incubated with DCFH-DA for 30 min at 37 °C in the dark. The intensity of fluorescence was then measured with an excitation wavelength of 488 nm and an emission wavelength of 525 nm using the SpectraMax Plus Reader (Molecular Devices, San Jose, United States).

#### Antioxidant defense

The activity of catalase (CAT) was determined spectrophotometrically. The samples were homogenised with a KPE buffer and centrifuged at 8,000 x g for 45 min at 4 °C. After this step, the samples were diluted in a 10x KPE buffer. Subsequently, 1 µL of the sample was added to 199 µL of a mixture of 50 mL distilled water and 80 µL H_2_O_2_. H_2_O_2_ consumption was measured at 230 nm, at 30 °C, using a Half Area UV Star plate, over a period of 4 min, using the SpectraMax Plus Reader (Molecular Devices, San Jose, United States).

### Statistical analysis

Data were expressed as mean ± standard error of the mean (SEM). Normality of data was tested using the Shapiro-Wilk test. Comparisons between groups were performed using two-way ANOVA followed by Tukey’s *post hoc* test for multiple comparisons to identify significant group differences. Statistical analyses were performed using GraphPad Prism version 8.0.1 (GraphPad Software Inc., La Jolla, United States). Differences were considered statistically significant at p < 0.05.

## Results

### Pyridoxamine attenuates metabolic parameters and liver damage in a diet-induced MASH model

To establish a preclinical model of MASLD, mice were fed either a standard diet (Control) or a high-fat, high-carbohydrate diet with the addition of 2% cholesterol (HFHC + CHOL2%) for 12 weeks. From the sixth to the 12th week, the mice in the Control and HFHC + CHOL2% groups were given pyridoxamine, while the other mice received the vehicle.

**FIGURE 1:**
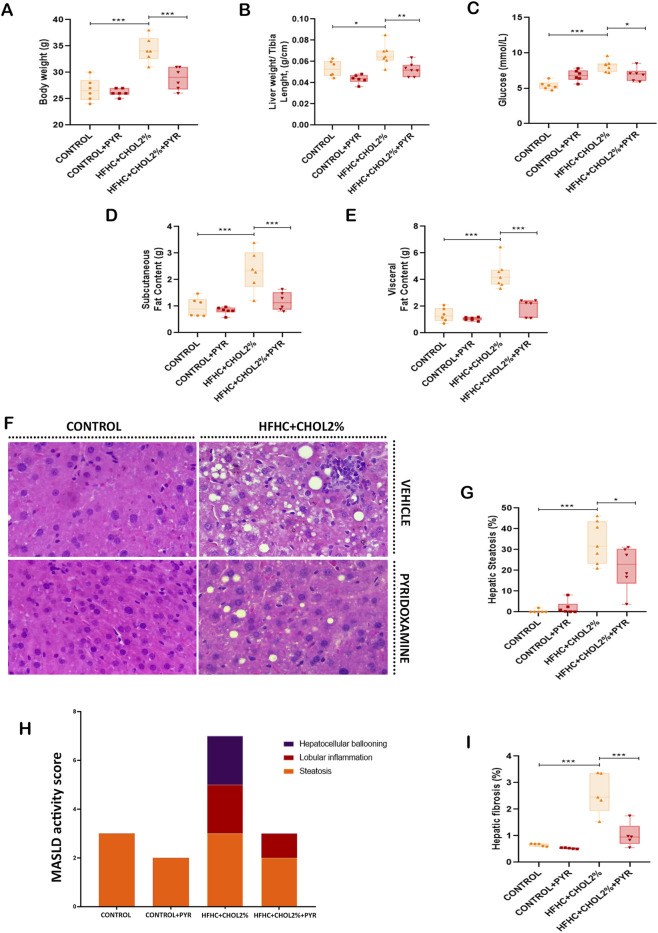
Pyridoxamine improves metabolic function and liver histopathological features in mice with diet-induced MASH. At the end of the experiment, body weight **(A)** and liver weight normalised to tibia length **(B)** were measured. Fasting glucose levels were determined after 6 h of fasting **(C)**. Adipose tissue distribution was evaluated, including subcutaneous **(D)** and visceral fat **(E)**. Representative H&E-stained liver sections illustrate hepatic histopathology **(F)**, and quantitative analyses include hepatic steatosis **(G)**, MASLD activity score **(H)**, and fibrosis area **(I)**. Data are presented as mean ± SEM (n = 5–8). Statistical significance: *P < 0.05, **P < 0.01, ***P < 0.001. Images were acquired at 20× magnification.

As detailed in Table 1, the HFHC + CHOL2% diet is characterized by a predominance of saturated fat and a reduced content of complex carbohydrates compared with the control diet. Consistent with its higher energy density, HFHC + CHOL2% feeding resulted in a significant increase in total caloric intake relative to the control diet. Pyridoxamine treatment did not reduce total caloric intake or solid food consumption in HFHC + CHOL2%-fed mice, but was associated with a significant reduction in water intake (Supplementary Figure S1). After 12 weeks of dietary exposure, body and liver weights were significantly increased in the HFHC + CHOL2% group compared with control animals, whereas pyridoxamine treatment in HFHC + CHOL2%-fed mice attenuated these increases (Figures 1A,B). Feeding of HFHC + CHOL2% resulted in an impairment of glycaemic homeostasis, as evidenced by a significant increase in fasting glucose levels compared to the control group. The administration of pyridoxamine in the HFHC + CHOL2% group significantly attenuated this increase (Figure 1C). In addition, the HFHC + CHOL2% group showed an increase in subcutaneous and visceral fat tissue compared to the control group, which was significantly reduced by pyridoxamine treatment in the HFHC + CHOL2% fed-mice (Figures 1D,E).

Hepatic histological analyses were performed to characterise the MASLD phenotype. Analysis of H&E staining showed a significant increase in steatosis in the HFHC + CHOL2% group compared to the control group. Conversely, HFHC + CHOL2%-fed mice treated with pyridoxamine showed significantly lower intracellular lipid accumulation than HFHC + CHOL2% group (Figures 1F,G). The MASLD activity score (NAS score) was applied in all groups, with the HFHC + CHOL2% group achieving a score of 7, corresponding to MASH classification, while the other groups scored between 2 and 3, indicating non-MASH status (Figure 1H). Analyses of liver fibrosis using Masson’s trichrome staining revealed greater collagen deposition in the HFHC + CHOL2% group compared to Control group. In contrast, administration of pyridoxamine in the HFHC + CHOL2% group significantly reduced collagen deposition compared to the HFHC + CHOL2% group (Figure 1I).

Biochemical analysis of the serum showed that the HFHC + CHOL2% group had significantly higher levels of total cholesterol, LDL, and HDL compared to the control group (Table 3). Pyridoxamine treatment effectively lowered total cholesterol and LDL concentrations in the HFHC + CHOL2% group while HDL concentrations remained unchanged compared to the HFHC + CHOL2% group (Table 3). No significant differences were observed in serum triglyceride levels among the groups (Table 3). Serum ALT levels were significantly increased in the HFHC + CHOL2% group compared to controls (Table 3). Hepatic cholesterol and triglyceride levels were also significantly higher in the HFHC + CHOL2% group compared to the control group. Importantly, treatment with pyridoxamine in the HFHC + CHOL2% fed-mice attenuated both hepatic lipid accumulation and serum ALT levels in mice fed HFHC + CHOL2% cholesterol (Table 3).

### Pyridoxamine protects against microcirculatory alterations in a diet-induced MASH model

**FIGURE 2:**
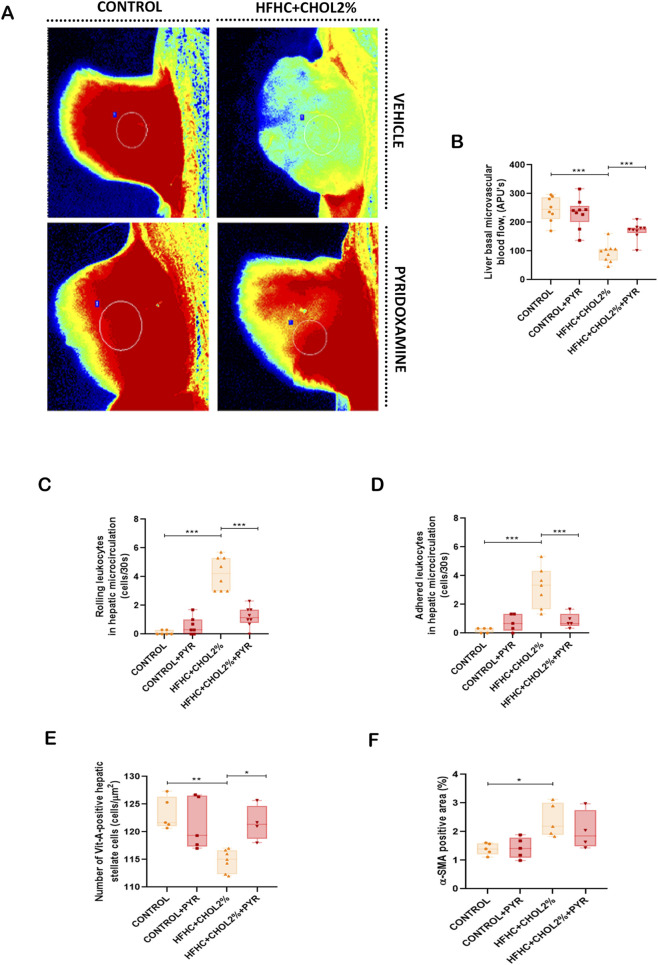
Pyridoxamine restores hepatic microvascular function and stellate cell activity in mice with diet-induced MASH. Basal liver blood flow was assessed by laser speckle flowmetry **(A,B)**. Leukocyte dynamics, including rolling **(C)** and adherent **(D)** leukocytes, were assessed by intravital microscopy. Vitamin A–positive hepatic stellate cell area **(E)** and α-SMA–positive area was evaluated by immunohistochemistry **(F)**. Data are presented as mean ± SEM (n = 5–8). Statistical significance: *P < 0.05, **P < 0.01, ***P < 0.001.

To investigate the effects of pyridoxamine on hepatic microcirculatory function, we used intravital microscopy and laser speckle contrast imaging (LSCI). The mice in the HFHC + CHOL2% group had significantly lower hepatic basal microvascular perfusion compared to the control group. Administration of pyridoxamine significantly improved hepatic perfusion in the HFHC + CHOL2%-fed mice (Figures 2A,B).

Activation of hepatic stellate cells (HSC) was more pronounced in the HFHC + CHOL2% group compared to the control group, as evidenced by a lower number of vitamin A-positive cells. Treatment with pyridoxamine in the HFHC + CHOL2% group restored the number of quiescent HSCs, indicating an attenuation of fibrogenic activation (Figure 2C). Accordingly, immunohistochemical analysis revealed an increase in α-SMA-positive areas in the HFHC + CHOL2% group, a marker of activated HSCs, which was significantly reduced by treatment with pyridoxamine in the MASH mouse model (Figure 2D).

In addition, leukocyte-endothelial interactions were significantly increased in the HFHC + CHOL2% group, as evidenced by a significant increase in rolling and adherent leukocytes in the sinusoids and post sinusoidal venules, compared with the control group. Treatment with pyridoxamine in the HFHC + CHOL2% fed-mice reduced the recruitment of leukocytes to the liver endothelium (Figures 2E,F).

### Pyridoxamine controls inflammation in a diet-induced MASH model

**FIGURE 3:**
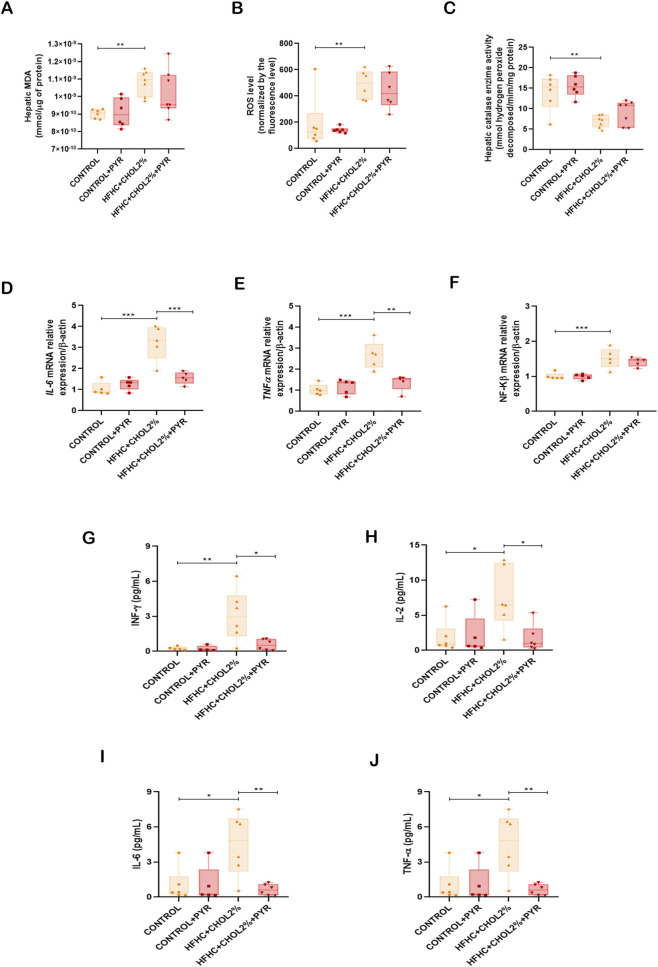
Pyridoxamine attenuates hepatic oxidative stress and inflammatory responses in mice with diet-induced MASH. Hepatic MDA levels were quantified using the TBARS assay **(A)**, ROS formation was assessed by DCFH-DA fluorescence **(B)**, and catalase (CAT) activity was measured spectrophotometrically **(C)**. Gene expression of IL-6, TNF-α, and NF-κB was evaluated by qPCR **(D–F)**, while protein levels of IL-6, TNF-α, IFN-γ, and IL-2 were quantified by CBA using flow cytometry **(G–J)**. Data are presented as mean ± SEM (n = 5–8). Statistical significance: *P < 0.05, **P < 0.01, ***P < 0.001.

Next, we analysed the hepatic markers of oxidative stress in MASH. The mice on the HFHC + CHOL2% diet showed a significant increase in malondialdehyde (MDA) and reactive oxygen species (ROS) levels compared to the control group. In addition, a reduction in hepatic catalase activity was observed in mice with MASH compared to the control group. Treatment with pyridoxamine did not alter these oxidative stress parameters (Figures 3A–C).

Given the increased leukocyte recruitment observed in MASH mice, we next analysed the cytokine profile in the liver of the mice treated or not with pyridoxamine. Gene expression analysis revealed that the HFHC + CHOL2% group had upregulation of key proinflammatory mediators, including IL-6, TNF-α and NF-κB, compared with the control group. Treatment with pyridoxamine in the HFHC + CHOL2% group significantly attenuated IL-6 and TNF transcriptional activation (Figures 3D–F). At the protein level, this inflammatory signature was confirmed by increased levels of TNF-α, IFN-γ, IL-6 and IL-2 in the HFHC + CHOL2% group compared to the control group and administration of pyridoxamine in the HFHC + CHOL2% group significantly reduced this inflammatory profile (Figures 3G–J).

### Pyridoxamine modulates the AGE/ALE-RAGE pathway in a diet-induced MASH model

**FIGURE 4:**
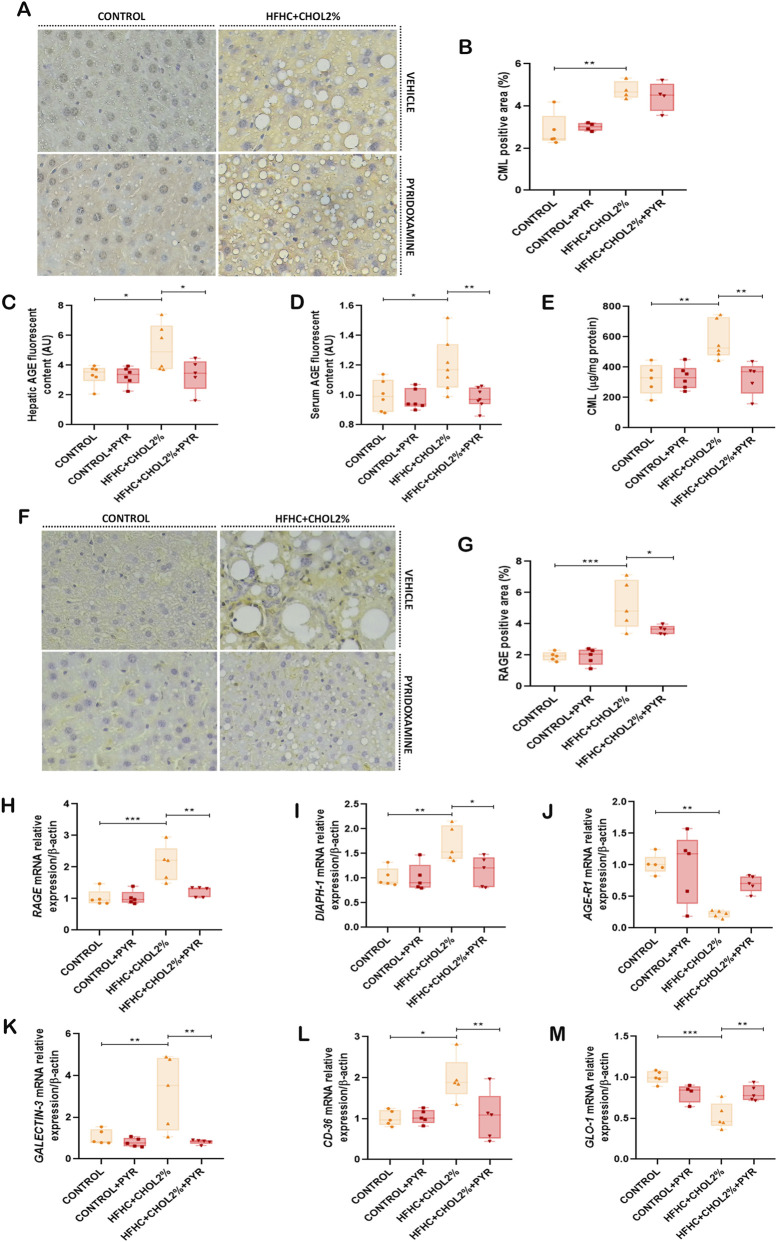
Pyridoxamine mitigates hepatic AGE accumulation and modulates AGE receptor expression in mice with diet-induced MASH. Representative immunohistochemical staining illustrates CML expression **(A)**, with quantitative analysis in **(B)**. Hepatic and serum fluorescent AGE concentrations were measured using a microplate reader at excitation/emission 360/460 nm **(C,D)**, and hepatic CML content was quantified by ELISA **(E)**. Representative immunohistochemical staining for RAGE is shown in **(F)**, with quantitative RAGE-positive area in **(G)**. Gene expression of RAGE, DIAPH-1, AGE-R1, Galectin-3, CD-36, and glyoxalase-1 (GLO-1) was evaluated by qPCR **(H–M)**. Pyridoxamine decreased AGE deposition and downregulated RAGE and related receptors while enhancing detoxifying pathways. Data are presented mean ± SEM (n = 5–8). Statistical significance: *P < 0.05, **P < 0.01, ***P < 0.001. Images were acquired at ×20 magnification.

To investigate the contribution of AGE/ALE metabolism to the development of MASH, immunohistochemical and ELISA quantifications showed a significant accumulation of CML in hepatic tissue of HFHC + CHOL2% mice compared to the control group, which was unaffected by pyridoxamine (Figures 4A,B,E). In contrast, analysis of fluorescent AGE levels determined by the Nakayama method showed a significant increase in both serum and liver of the HFHC + CHOL2% fed-mice, which was reduced by pyridoxamine treatment in the HFHC + CHOL2%-fed mice (Figures 4C,D).

Next, we examined the hepatic expression of AGE receptors and detoxification systems. RAGE was significantly upregulated in the HFHC + CHOL2% group compared with the control group, as determined by both immunohistochemistry and gene expression analyses. In addition, gene expression analysis revealed an increase in DIAPH-1, a downstream adaptor protein that mediates RAGE-dependent intracellular signalling. Treatment with pyridoxamine in the HFHC + CHOL2% group normalised RAGE expression at both the gene and protein levels, as well as DIAPH-1 gene expression, to levels comparable to those of the control group (Figures 4F–I).In parallel, the HFHC + CHOL2% group showed altered expression of other AGE/ALE-related receptors: the expression of AGE-R1 was suppressed, while the expression of galectin-3 (GAL-3) and CD36 was significantly increased compared to the control group. Treatment with pyridoxamine in the HFHC + CHOL2% fed-mice restored AGE-R1 levels and decreased the expression of GAL-3 and CD36 (Figures 4J–L).

The detoxification enzyme glyoxalase-1 (GLO-1), an important regulator of MGO metabolism, was reduced in the HFHC + CHOL2% group compared to the control group. Treatment with pyridoxamine in the HFHC + CHOL2% group restored hepatic GLO-1 expression to control levels (Figure 4M).

**FIGURE 5:**
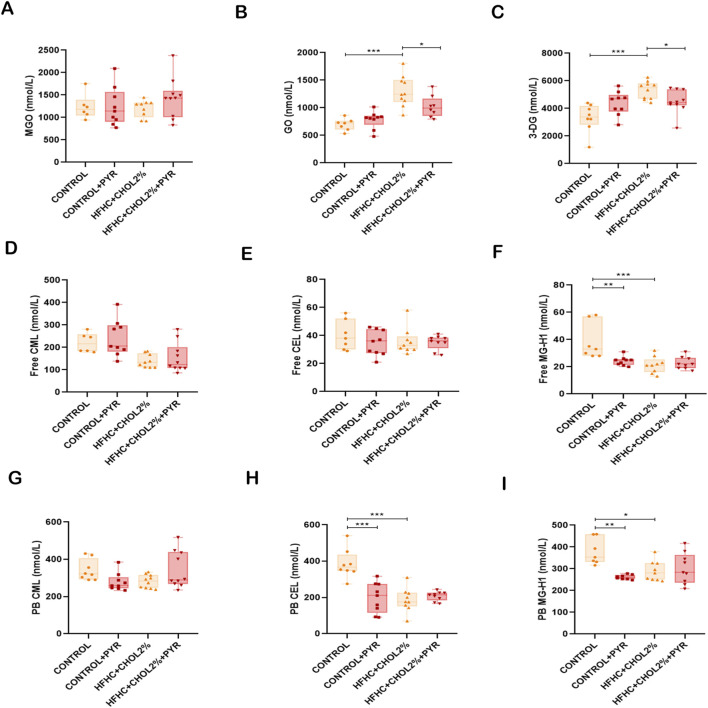
Pyridoxamine reduces circulating AGEs and reactive intermediates in mice with diet-induced MASH. Serum levels of MGO, GO, and 3-DG were measured by UPLC-MS/MS **(A–C)**. Free CML, CEL, and MG-H1 **(D–F)**, as well as protein-bound CML, CEL, and MG-H1 **(G–I)**, were quantified by UPLC-MS/MS. Pyridoxamine lowered systemic levels of reactive dicarbonyls and AGE compounds compared to untreated HFHC + CHOL2% mice. Data are presented as mean ± SEM (n = 5–8). Statistical significance: *P < 0.05, **P < 0.01, ***P < 0.001.

We quantified serum levels of dicarbonyls and AGE by UPLC-MS/MS. While plasma levels of MGO remained unchanged in the groups (Figure 5A), mice in the HFHC + CHOL2% group showed a significant increase in glyoxal (GO) and 3-deoxyglucosone (3-DG) compared to the control group. Treatment with pyridoxamine in the HFHC + CHOL2% group effectively attenuated these increases, indicating inhibition of early glycation intermediates (Figures 5B,C).

In contrast, the HFHC + CHOL2% group showed significantly lower levels of free CML and MG-H1, as well as protein-bound CEL and MG-H1, compared to the control group (Figures 5F,H,I). Pyridoxamine treatment did not significantly alter these reductions in HFHC + CHOL2%-fed mice (Figures 5D–I); however, it reduced free MG-H1 levels and protein-bound CEL and MG-H1 levels in control-fed mice (Figures 5F,H,I).

## Discussion

As increasing evidence suggests that the AGE/ALE pathway can contribute to MASLD progression and new pharmacological interventions for MASH are urgently needed, we aimed in this study to explore how pyridoxamine is associated with modulation of hepatic AGE-related signaling at multiple levels in a diet-induced murine model of MASH by assessing key metabolites and receptors of the pathway, as well as its pharmacological impact on key features of MASH, including metabolic, inflammatory, microcirculatory, and oxidative stress parameters. Importantly, the HFHC + CHOL2% dietary model was intentionally selected to reproduce a Western-style nutritional pattern, characterized by the combined intake of saturated fat, cholesterol, and refined sugars. This approach enhances translational relevance, as these dietary components typically coexist and act synergistically in the clinical context of MASLD/MASH.

Pyridoxamine was associated with a marked attenuation of hepatic and systemic metabolic imbalance and ameliorated key MASLD hallmark features. Specifically, pyridoxamine reduced fasting blood glucose levels, body weight, visceral and subcutaneous fat, liver weight, hepatic steatosis, circulating and hepatic cholesterol levels, hepatic triglyceride levels, and ALT activity. These metabolic alterations occurred in the context of significantly increased total caloric intake induced by the HFHC + CHOL2% diet. Importantly, these metabolic improvements also translated into histopathological benefits as pyridoxamine-treated mice exhibited lower NAS scores, indicating attenuation of MASH progression in parallel with metabolic improvement. These effects are consistent with the ability of pyridoxamine to suppress hepatic lipogenesis and improve insulin sensitivity as demonstrated by previous studies (Liu et al., 2016; Maessen et al., 2016; Alshanwani et al., 2022; Van den Eynde et al., 2021). Although the ability of pyridoxamine to modulate the AGE/ALE-RAGE axis in different pathological contexts has been extensively studied (Pereira A. et al., 2017; Hagiwara et al., 2009; Mahdavifard and Hasani, 2025), our findings provide new evidence that pyridoxamine is associated with modulation of the AGE/ALE axis at multiple levels in MASH. Oral administration of pyridoxamine significantly reduced systemic levels of reactive dicarbonyls, such as glyoxal (GO) and 3-deoxyglucosone (3-DG), and prevented the accumulation of fluorescent AGE/ALEs and CML in both serum and liver. It should be noted that methylglyoxal (MGO) was assessed exclusively in the circulation, and hepatic MGO levels were not directly measured in this study. Given that MGO is predominantly generated intracellularly and rapidly detoxified, circulating MGO may underestimate tissue-specific accumulation. In this context, the observed reduction in hepatic (GLO-1) mRNA expression in HFHC + CHOL2%-fed mice suggests impaired intracellular detoxification capacity, supporting the notion that unchanged serum MGO levels do not preclude increased tissue-level carbonyl stress. This distinction is particularly relevant given that glycative and lipoxidative stress are increasingly recognized as key mediators of hepatocellular injury in MASH, linking metabolic imbalance to persistent cellular dysfunction (Dorairaj et al., 2021; Leung et al., 2016). In addition, pyridoxamine was associated with modulation of key components of the hepatic AGE/ALE signalling pathway by downregulating receptors such as RAGE, CD36, and galectin-3, while upregulating detoxification-related mediators, including AGE-R1 and GLO-1. This coordinated rebalancing of receptor expression and detoxification pathways is consistent with attenuation of the self-perpetuating AGE/ALE–RAGE signaling loop implicated in MASH progression (Wouters et al., 2021; Hollenbach et al., 2017; Hollenbach, 2017). In contrast, GO and 3-DG arise from multiple metabolic routes linked to lipid overload, oxidative stress, and enhanced degradation of Amadori intermediates, which may preferentially promote their systemic accumulation under HFHC + CHOL2% feeding conditions.

These results extend our previous work on MASLD models by confirming that interventions targeting AGE/ALE-RAGE accumulation are associated with attenuation of MASH features (Pereira et al., 2020; Pereira et al., 2021b). At molecular level, Zhang et al. (2024) have shown that RAGE blockade attenuates lipid accumulation in hepatocytes by suppressing SREBP-1c via inhibition of p65 NF-κB and p38 MAPK phosphorylation (Zhang et al., 2024). Consistently, Maessen et al. (2016) showed that pyridoxamine suppresses adipogenesis *in vitro* by reducing lipid accumulation, triglyceride content, and expression of adipogenic genes such as PPARγ, C/EBPα, and SREBP-1C (Maessen et al., 2016).

An important finding of this study is the ability of pyridoxamine to restore hepatic microcirculatory dynamics in the MASH model. Intravital microscopy and LSCI analyses showed was associated with improved endothelial function and enhanced microvascular perfusion, which is consistent with previous reports of reduced vascular stiffness and improved tissue perfusion after pyridoxamine treatments (Pereira et al., 2021b; Maessen et al., 2016; Reeve et al., 2023; Jiang et al., 2011; Van den Eynde et al., 2023; Van den Eynde et al., 2021). In addition, presently, pyridoxamine exerted potent anti-inflammatory effects in the context of MASH: besides its role as an inhibitor of AGE formation, pyridoxamine significantly reduced hepatic expression and secretion of key proinflammatory mediators, including TNF-α, IL-6, IFN-γ, and IL-2, in parallel with reduced NF-κB activation. This immunomodulatory effect was accompanied by a marked reduction in leukocyte recruitment in the hepatic microvasculature, suggesting suppression of endothelial activation and immune cell infiltration. These data support the concept that pyridoxamine is associated with attenuation of inflammatory responses at multiple levels, i.e., by limiting the accumulation of AGE/ALE that trigger receptor-mediated inflammatory signalling and by attenuating downstream cytokine cascades (Pereira et al., 2021b; Maessen et al., 2016; Oh et al., 2019). Furthermore, the anti-inflammatory effect of pyridoxamine may be closely related to its role in modulating the bioavailability of vitamin B6. Inflammation leads to tissue-specific degradation of pyridoxal 5′-phosphate (PLP), particularly in the liver, where it is diverted into immunometabolic pathways. Thus, the vitamin B6 deficiency can exacerbate inflammatory responses. Pyridoxamine may attenuate inflammation in MASH by a mechanism supported by evidence of PLP’s role in suppressing inflammasome activation and inflammatory signalling via TLR/NF-κB and JNK pathways (Ueland et al., 2017; Ueland et al., 2015; Chiang et al., 2005).

In parallel, pyridoxamine treatment was associated with reduced fibrotic remodeling of the liver, including lower collagen deposition and decreased markers of HSCs activation, a hallmark of fibrosis (Friedman, 2008). These antifibrotic effects are supported by previous studies showing that pyridoxamine downregulates the expression of fibrogenic mediators such as MMPs and collagen IV (Alshanwani et al., 2022; Goldin et al., 2006). Of note, the ability of pyridoxamine to maintain the integrity of the extracellular matrix may be linked to reduced cross-linking of AGE and ALE with structural proteins, thereby limiting matrix stiffening and fibrosis progression (Goldin et al., 2006). Taken together, these results highlight the therapeutic relevance of pyridoxamine in MASH, as its ability to attenuate inflammation and preserve extracellular matrix integrity directly counteracts the chronic, unresolved inflammatory milieu that drives hepatocellular injury, hepatic stellate cell activation, and fibrogenesis (Schwärzler et al., 2024).

Despite the well-documented antioxidant properties of pyridoxamine, in our study, pyridoxamine treatment did not significantly improve oxidative stress parameters in the liver. The concentrations of malondialdehyde (MDA) and reactive oxygen species (ROS) remained elevated, while catalase activity was persistently reduced in HFHC + CHOL2% mice. These results suggest that excessive cholesterol intake exceeds the antioxidant capabilities of pyridoxamine, which is consistent with previous findings indicating that a hypercholesterolaemic diet disrupts endogenous antioxidant defences and exacerbates oxidative damage (Jiang et al., 2021; Alipour et al., 2006). Furthermore, as MASLD progresses to MASH, endogenous antioxidant reserves are gradually depleted, reducing the liver’s ability to counteract oxidative stress (Pereira et al., 2020; Pereira et al., 2022; Pereira E. N. G. S. et al., 2017; Koumenis et al., 2002). In our study, the improvement in hepatocellular injury appeared to result from reductions in steatosis, fibrosis, and inflammation, rather than from modulation of oxidative stress, underscoring the central role of these pathological processes in restoring liver function.

Some limitations of the present study are now acknowledged. First, although the improvements associated with pyridoxamine in metabolic, inflammatory, microcirculatory, and fibrotic parameters occurred in parallel with modulation of the hepatic AGE/ALE–RAGE axis, the absence of receptor-blocking approaches, genetic models, or cell-based experiments precludes definitive causal attribution to this pathway. Second, the AGE content of the experimental diets was not directly quantified, limiting the ability to discriminate between the relative contributions of dietary-derived AGEs and endogenously formed AGEs to the observed effects. In addition, reactive dicarbonyls, including MGO, GO, and 3-DG, were assessed exclusively in the circulation, and their hepatic levels were not directly quantified. Finally, as with all preclinical studies, extrapolation of these findings to human MASLD/MASH should be undertaken with caution due to species-specific differences in metabolism and disease progression.

Our results indicate that pyridoxamine counteracts inflammatory, metabolic, and microvascular disturbances in a diet-induced pre-clinical model of MASH in association with modulation of the hepatic AGE/ALE axis at multiple levels. These findings further reinforce that the hepatic AGE signaling pathway plays a significant role in MASH pathophysiology and provide new mechanistic insight into the exact mechanisms of pyridoxamine-mediated liver protection.

## Data Availability

The authors acknowledge that the data presented in this study must be deposited and made publicly available in an acceptable repository, prior to publication. Frontiers cannot accept a manuscript that does not adhere to our open data policies.

## Supplementary material

SUPPLEMENTARY FIGURE S1 Water intake, food intake, and total caloric intake in mice fed control or HFHC+CHOL2% diets. Throughout the experimental period, solid food intake (A), water intake (B), and total caloric intake (C) were monitored weekly at the cage level. HFHC+CHOL2%-fed mice exhibited reduced solid food consumption and increased water intake compared to control animals, resulting in a significantly higher total caloric intake. Data are presented as mean ± SEM (n ~ 5 animals per cages). Statistical significance: *P < 0.05, **P < 0.01, ***P < 0.001.
