# Supplementary figures and images for: Pyridoxamine reduces inflammatory and microcirculatory abnormalities in metabolic dysfunction-associated steatohepatitis and modulates key factors in the hepatic AGE/ALE signaling pathway
Source: Front Physiol. 2026 Jan 15;16:1736221. doi: 10.3389/fphys.2025.1736221 (PMC12852023; doi:10.3389/fphys.2025.1736221)

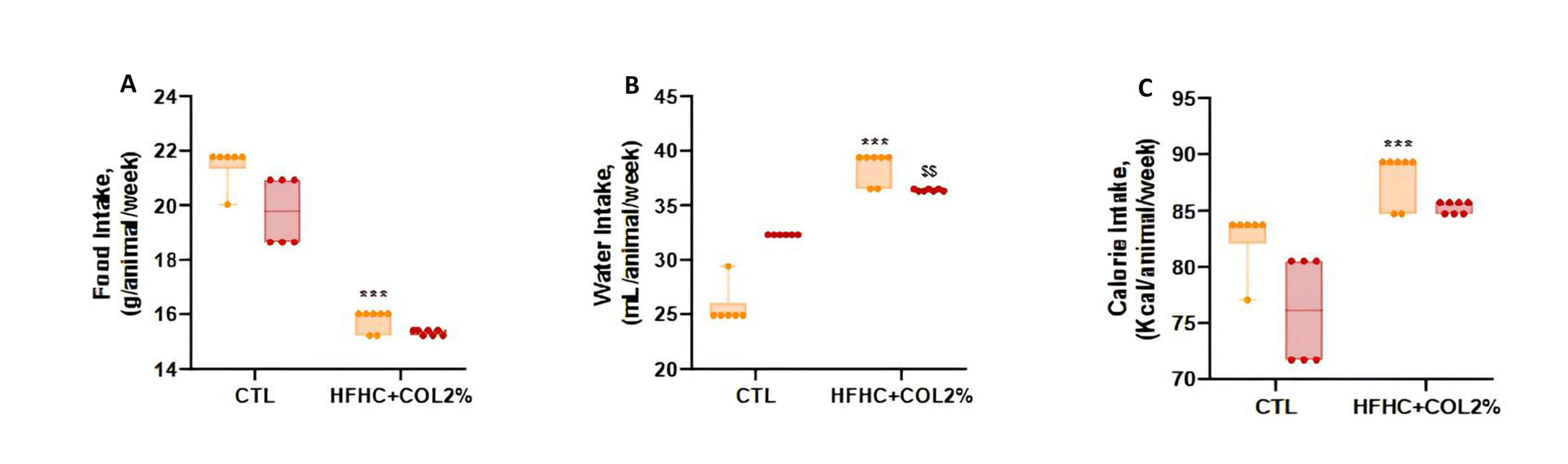

Supplement: Supplementary file 1 [file Image1.tif]
